# Imaging of Increasing Damage in Steel Plates Using Lamb Waves and Ultrasound Computed Tomography

**DOI:** 10.3390/ma14175114

**Published:** 2021-09-06

**Authors:** Monika Zielińska, Magdalena Rucka

**Affiliations:** 1 Department of Technical Fundamentals of Architectural Design, Faculty of Architecture, Gdańsk University of Technology, Narutowicza 11/12, 80-233 Gdańsk, Poland; 2 Department of Mechanics of Materials and Structures, Faculty of Civil and Environmental Engineering, Gdańsk University of Technology, Narutowicza 11/12, 80-233 Gdańsk, Poland; magdalena.rucka@pg.edu.pl

**Keywords:** non-destructive testing, steel plates, ultrasonic tomography, damage detection

## Abstract

This paper concerns the inspection of steel plates, with particular emphasis on the assessment of increasing damage. Non-destructive tests were performed on four plates, one of which was undamaged, while the remaining three had defects in the form of circular holes with diameters of 2, 5 and 10 cm. Guided Lamb waves were used in the research, and the image reconstruction was performed using ultrasound computed tomography. The damage size was estimated by tracking the real course of rays and densifying the pixel grid into which the object was divided. The results showed the great potential of ultrasound tomography in detecting defects in steel elements, together with the possibility of estimating damage size.

## 1. Introduction

Imaging potential defects and their exact position and size are among the greatest challenges of recent studies in non-destructive testing (NDT) and structural health monitoring (SHM). One of the most efficient techniques is to use ultrasonic waves followed by their processing. In general, two approaches are possible for damage imaging. In the first, the guided wave field is sensed over an inspected area (usually in a non-contact manner) and it is then subjected to further processing using, for example, root mean squares to obtain a useful defect image [1,2]. The second approach is connected with the use of an array of piezoelectric transducers, acting as both actuators and sensors, attached to selected points on an analysed structure. Wave propagation signals are collected and then processed using appropriate damage detection algorithms such as the time of flight (TOF) based triangulation method [3], supported by ellipse or hyperbolic probability imaging [4,5], the time-reversal technique [6], the migration technique [7], phased-array beamforming [8] and finally ultrasound tomography, widely used for inspection of structures made of concrete [9,10,11], metal [12,13,14] and composite materials [15,16].

Tomography using guided ultrasound waves has become a popular technique incorporated into the structural health monitoring of elements of civil engineering infrastructure such as plates, shells and pipes [17,18,19,20,21,22], and also in the monitoring of aircraft components [23]. The increasing use of non-destructive methods for detecting defects in plate elements has resulted in a growing need to increase the effectiveness and efficiency of inspection. When performing tests based on Lamb waves, some practical problems affecting the results may be encountered. These problems are discussed extensively in the literature. Rao et al. [24] carried out a study on monitoring the corrosion of steel plates and the reconstruction of the thickness of corrosion damage. Wang et al. [25] used guided waves to image a hole with a corroded edge. Zhao et al. [26] investigated the imaging of damage in aluminium plates, comparing different tomography algorithms. Leonard et al. [27] analysed Lamb wave tomography on both aluminium and composite plates, with defects of various sizes and thicknesses. The possibility of imaging defects in the form of round and rectangular holes in aluminium and composite plates was tested by Khare et al. [28]. Balvantin and Baltazar [29] analysed the images of an aluminium plate containing damage in the form of a two-stage circular discontinuity, using the multiplicative algebraic reconstruction technique (MART) and back projection. The structural condition monitoring system for composite panels with openings was presented by Prasad et al. [30]. The size of the defect itself is also of great importance in the imaging of tomographic plates, as evidenced by Menke and Abbott [31], Cerveny [32] and Belanger and Cawley [33].

The research described in this article aims to evaluate the use of ultrasound tomography to locate surface damage of varying sizes. The experimental and numerical analyses were carried out on four steel plates, one intact and three containing defects in the form of circular holes with diameters of 2, 5 and 10 cm. The non-destructive inspection was carried out using Lamb waves and ultrasound tomography (UT). The reconstruction of the Lamb wave propagation velocity was performed using both the non-reference approach and a method based on the differences in the transition times between the reference model and the three damaged models with surface defects. In order to improve the image quality, certain methods of tracing the real course of wave paths were used. The influence of mesh density on the possibility of estimating damage size was also assessed.

## 2. Ultrasound Tomography—Theoretical Background

**Figure 1:**
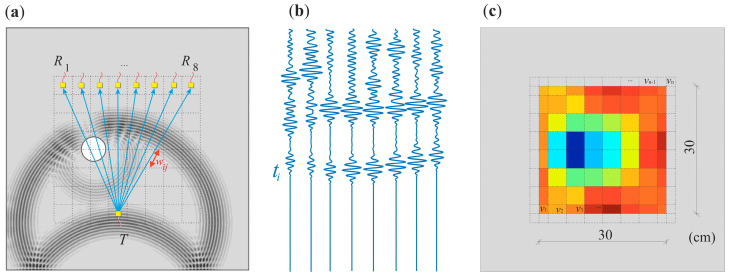
Schematic diagram of velocity reconstruction using ultrasound tomography: (**a**) plate with indicated receivers (*R*), transmission (*T*) points and simulated wave field; (**b**) guided wave propagation time signals; (**c**) ultrasound tomography map.

Ultrasound tomography imaging allows recreating the internal structure of an examined object using the properties of elastic waves. A schematic process of performing ultrasound tomography is shown in Figure 1. The first step is to divide the test sample into cells called pixels. Each one of them constitutes a discrete area in the tested element. Such a division is shown with a dashed line in Figure 1a. Then, after applying appropriate algorithms, each pixel takes a value representing the speed of the ultrasonic wave propagated through this area.

Image reconstruction is based on information from ultrasound wave signals propagating from transmitting points (*T*) to receiving points (*R*). This information may be, for example, the time of flight (TOF), measured along with the multiple ray paths, determined after appropriate signal processing [34,35,36]. Each obstacle in the path of the travelling wave changes the propagation time. Among them, we can distinguish defects that delay the wave reaching the receiver, such as air voids or cracks, and those that decreased total velocity along all rays passing through these inclusions. The latter include inclusions made of materials whose propagation velocity is higher than in the surrounding medium. Based on the time of flight of the wave, with the known geometry of the tested object, it is possible to determine wave propagation speed, correlated with the mass density, modulus of elasticity, and Poisson’s ratio characteristic for a given material.

The time of flight between the transmitter (*T*) and the receiver (*R*) can be described by a line integral of the transition time distribution along the propagation way, *w*:

(1) $$t=\int_{0}^{w_{1}}dt=\int_{0}^{w_{1}}sdw=\int_{0}^{w_{1}}\frac{1}{v}dw,$$

where *v* is the average velocity, $w_{1}$ denotes the distance between transmitter and receiver and *s* is the inverse of the velocity, v, referred to as slowness, s=1/v. Measurement of the wave travel time along specific paths enables the determination of local velocities in the tested plate. The wave velocity for each cell, $v_{j}$, along the path of the wave from the transmitter to the receiver can be determined from the following formula:

(2) $$t_{i}=\sum_{j=1}^{n}w_{ij}s_{j},\;\;i=1,2,3,\ldots ,m,\;\;j=1,2,3,\ldots ,n,$$

where *m* denotes the number of rays, *n* denotes the number of pixels into which the tested sample is divided, $t_{i}$ is the time of flight of the guided wave along the *i*-ray, $w_{ij}$ is the *i*-ray propagation path through the *j*-pixel and $s_{j}$ is the slowness at pixel *j*. It is assumed that velocity, $v_{j}$, in individual cells is constant.

One of the techniques for solving this type of system of equations is the algebraic reconstruction technique (ART) [37]. It was used to perform calculations in the conducted research. First, each cell is assigned the same slowness. Its value is calculated as the inverse of the average velocity of ultrasonic wave propagation in the tested material. Based on the initial image created in this way, the iteration process begins and corrections are made.

## 3. Materials and Methods

### 3.1. Description of Specimens

**Figure 2:**
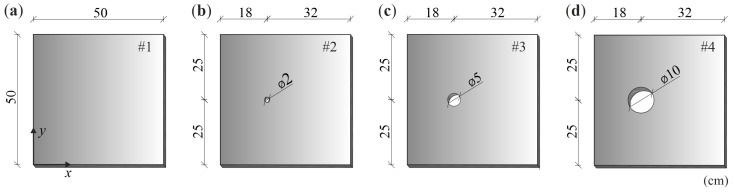
The geometry of the tested plates: (**a**) plate without damage (#1); (**b**–**d**) plates with surface damage (#2–#4).

**Figure 3:**
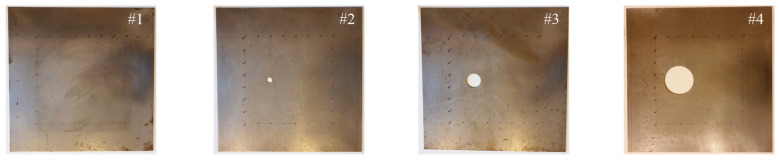
Photographs of tested plates (models #1–#4).

The tests were carried out on four steel plates with dimensions of 50 cm × 50 cm and 3 mm thickness. A reference model was the first element without damage (plate #1). The other three plates contained a circular hole of variable diameter: 2 cm (plate #2), 5 cm (plate #3) and 10 cm (plate #4), which represented damage of a surface type. Holes were made through the entire thickness of the plate. The centres of all holes were placed symmetrically in the *y*-axis direction and at a distance of 18 cm from the outer edge of the plate in the *x*-axis direction. The geometry of the test models is shown in Figure 2. Photographs of the plates used for the experimental tests are presented in Figure 3.

### 3.2. Experimental Investigations

**Figure 4:**
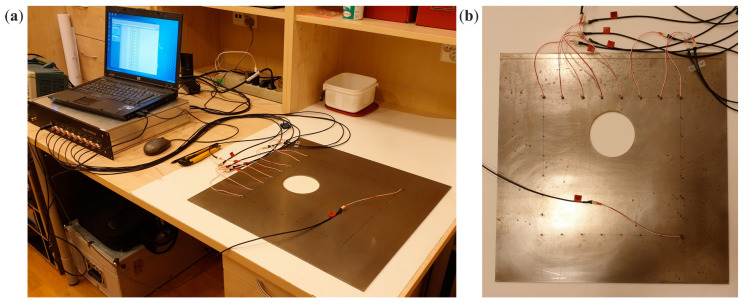
Experimental setup (**a**) and view on the steel plate with piezoelectric transducers (**b**).

Experimental tests of the propagation of Lamb waves in the steel plates were performed using a system for generation and registration of ultrasonic waves, the PAQ-16000D system (EC Electronics, Krakow, Poland). The experimental setup is shown in Figure 4. For both transmitting and receiving Lamb wave signals, plate piezoelectric transducers, NAC2024 (Noliac, Kvistgaard, Denmark), were applied. The excitation was a wave packet with a central frequency of 150 kHz, consisting of five sinusoid cycles modulated with the Hann window.

**Figure 5:**
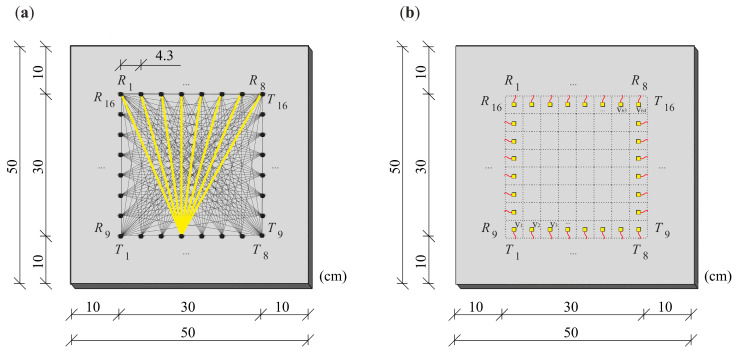
The plate with the marked inspected area: (**a**) location of transmitters *T*_1_–*T*_16_ and receivers *R*_1_–*R*_16_, (**b**) division of the area into pixels.

The inspected region of the plate was a square with dimensions of 30 cm × 30 cm, with a margin of 10 cm from each of the outer edges of the plate. The excitation was applied at 16 points (8 points on two perpendicular edges), and wave propagation signals were also sensed at 16 points. The transmitting points are marked in Figure 5a as *T*_1_–*T*_16_, and the receiving points as *R*_1_–*R*_16_. During measurements, the transmitter was placed at a specific point, and the output signals were registered at 8 points located on the opposite edge. The transmitter was then moved to the next point, and the measurement was repeated. Altogether, 128 wave propagation time signals were collected.

The image reconstruction process started by dividing the inspected region of the plate into 64 pixels. The number of pixels was derived from the number of measurement points located on each edge of the plate (8 × 8 points). Each pixel had a dimension of 4.3 cm × 4.3 cm. Pixels are marked with dashed lines in Figure 5b. Image reconstruction was made according to the collected signals of the wave transition from transmitters to receivers. The time of flight (TOF) of the ultrasonic wave for each of the *T*–*R* paths was determined. The tomography velocity image was created in MATLAB^®^ (9.3.0.713597, The MathWorks, Inc., Natick, MA, USA), based on the ART method. In the first step, it was assumed that the wave paths propagating from the transmitters to the receivers are straight. In the next step, the actual course of the rays was traced.

### 3.3. Identification of Material Parameters

**Figure 6:**
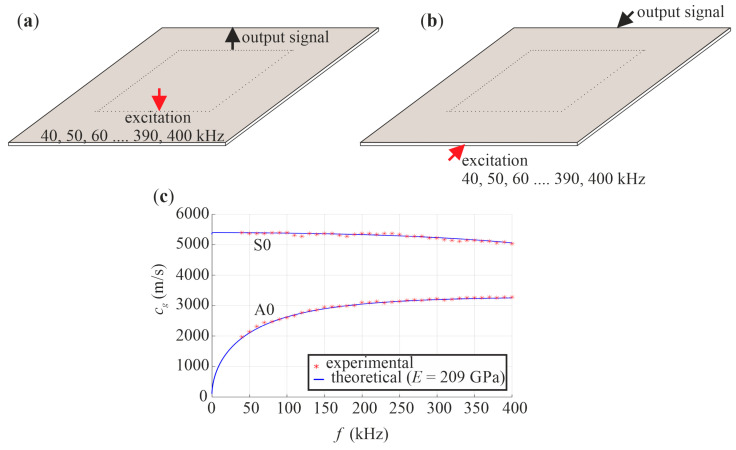
The scheme of experimental tests for determination of dispersion curves for (**a**) A0 mode and (**b**) S0 mode and (**c**) obtained dispersion curves.

Identification of material parameters was carried out for an intact plate #1. The experimentally determined mass density was ρ = 7893 kg/m^3^. Poisson’s ratio was set as 0.3. The dynamic elastic modulus was also determined experimentally based on a comparison of experimental and numerical dispersion curves. At first, dispersion curves of Lamb waves of two basic modes, i.e., symmetric S0 and antisymmetric A0 were determined. For this purpose, guided waves of frequencies ranging from 40 kHz to 400 kHz with a step of 10 kHz were excited and measured in two configurations, shown in Figure 6a,b. Theoretical dispersion curves were then calculated for different values of elastic modulus. Finally, the dynamic elastic modulus was determined by the method of least squares to give the best fit experimental and numerical modes, and its value was found to be 209 GPa (Figure 6c). The obtained values of the material parameters were then used for numerical simulations.

### 3.4. Numerical Modelling

Numerical analysis was carried out using the commercial Abaqus/Explicit program (ver 6.14, Dassault Systemes, Vélizy-Villacoublay, France) based on the finite element method (FEM). Four FEM models were prepared corresponding to the tested plates. Plate models were discretized with S4R elements with maximum dimensions of 1 mm × 1 mm. The boundary conditions were implemented free on all edges. The length of the integration step was 10^−7^ s. The excitation signal was the same as in the experiment, i.e., the 5-cycle wave packet of 150 kHz frequency, induced perpendicularly to the surface of the plate at points *T*_1_–*T*_16_. The output acceleration signals were collected at points *R*_1_–*R*_16_.

## 4. Results and Discussion

This research aimed to investigate the influence of increasing surface damage on the obtained tomographic maps. The analysis of the results of experimental and numerical studies was divided into four parts. The first one included tomographic imaging performed for the non-reference approach, with respect to time of transition between transmitters and receivers for each of the tested plates independently. The next part of the analysis consisted of preparing maps based on the differences in the transition times between the reference model and the three damaged models with surface defects. Then, the influence of the ray-tracing technique, taking into account the possibility of wave refraction, reflection and deflection was analysed. The final part focused on analysing the influence of the pixel mesh density on the possibility of estimating damage size.

### 4.1. Non-Reference Velocity Reconstruction

**Figure 7:**
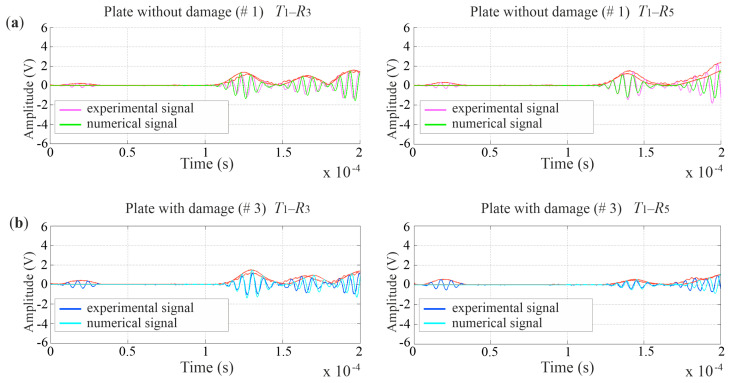
Comparison of selected Lamb wave signals for experimental and numerical tests in a plate without damage (**a**) and with damage (**b**).

Numerical and experimental signals transmitted and registered at selected points were the basis of tomographic maps. The comparison of selected Lamb wave signals for the experimental and numerical models is presented in Figure 7. The graphs show a high convergence between the results from both models.

**Figure 8:**
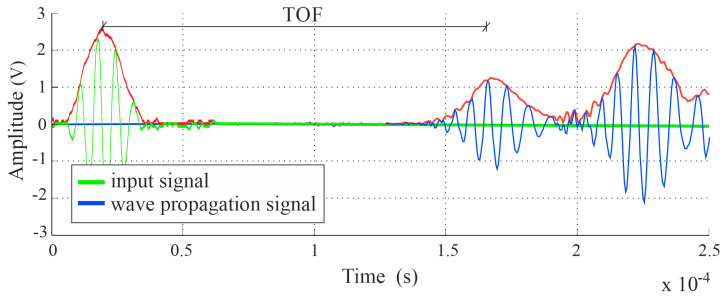
Determination of the time of flight (TOF) between transmitters and receivers.

The scheme of determining the time of flight (TOF) is shown in Figure 8. The estimation of this value is based on the first wave packet. The wave propagation time was established through the peak-to-peak method and was the difference between the peak value of the first wave pack of the output signal and the peak value of the input signal. The Hilbert transform was employed to create the signal envelope, which enabled the identification of peaks.

**Figure 9:**
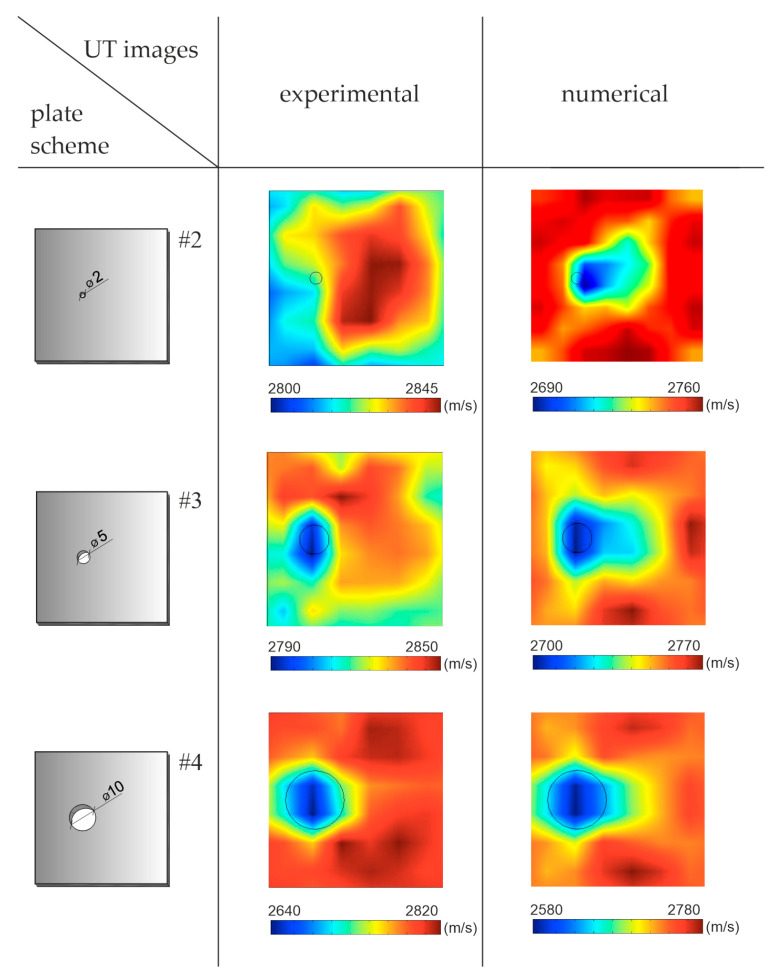
Ultrasonic tomography (UT) velocity maps using direct wave transition in plates with surface damage.

The ultrasonic tomography maps are illustrated in Figure 9. The first column shows the plate scheme; the next two present the tomographic maps based on experimental and numerical signals, respectively. The tomograms are constructed, based on the direct measurement of the wave travel time, which means that the results were not compared with the results obtained for the undamaged plate. Each of the performed tomograms is presented separately, ranging from the minimum to the maximum speed. The maps present results for all considered plates, with the increasing surface damage in the form of holes with a diameter of 2, 5 and 10 cm. The defect with a diameter of 2 cm was properly imagined only for data obtained from numerical tests. On the other hand, the holes with a diameter of 5 and 10 cm were successfully detected in both experimental and numerical results. The locations of this damage are marked as areas with a reduced speed in relation to the surrounding material. However, the size of the holes was difficult to estimate accurately.

The quantitative analysis of the values of wave propagation velocities is presented in Table 1 and Table 2 for the experimental and numerical data, respectively. The values of minimum, maximum and the mean of the wave velocity are given for all inspected plates. Table 1 and Table 2 also provide measures of variation in the form of standard deviation (SD) and coefficient of variation (CV). The wave velocities were calculated along all 128 paths propagating through the plate. The average velocity values for the experimental results (Table 1) were similar and ranged between 2791.01 m/s and 2835.33 m/s. These values apply to the plate with a 10 cm diameter defect and the undamaged plate, respectively. It is worth noting that the differences between the maximum and minimum velocity increased with increasing damage dimensions. This is because the increasing discontinuity of the material reduced the minimum velocity as the rays had to avoid damaged areas. The standard deviation increased with the growing damage area. A similar relationship was observed for the coefficients of variation, which took values ranging between 0.80% and 1.91%.

Table 2 gives the results of the quantitative analysis carried out for the numerical simulations. The average value of the wave propagation velocity varied between 2743.33 m/s and 2756.65 m/s. As in the case of results from the experimental studies, the difference between the maximum and minimum speed increased with the increasing damage area. The standard deviation was between 29.22 m/s and 51.36 m/s, while the variation coefficient ranged from 1.06% to 1.87%. These values indicate slight differences in the wave velocity along the path of the examined rays and the high sensitivity of ultrasound tomography to the occurrence of a defect.

### 4.2. Velocity Reconstruction with the Reference to Undamaged Plate

**Figure 10:**
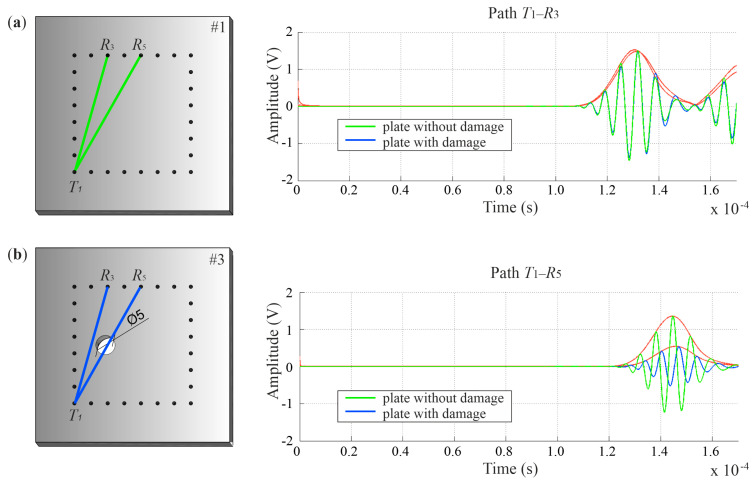
Comparison of wave signals propagated in intact plate (**a**) and plate with a 5 cm hole (**b**) along traces *T*_1_–*R*_3_ and *T*_1_–*R*_5_.

Ultrasound tomography often consists of comparing the data obtained for an undamaged structure with a damaged structure [38]. During health monitoring, as the damage size grows, the differences become more significant, and the intensity of changes in tomography images indicate the location of the damage. When comparing ultrasonic signals registered in a structure in the healthy (reference) and damaged (current) states, it is possible to indicate a difference in the TOF, especially if there is a defect along the inspected path. In the case of damage to the entire thickness of the element, the recorded signals travel along the path around the damage [39] so the paths are curved. Examples of Lamb wave signals propagated in an intact plate and a plate with a 5 cm hole along paths *T*_1_–*R*_3_ and *T*_1_–*R*_5_ are compared in Figure 10. When the wave passes through the hole (path *T*_1_–*R*_5_), its amplitude and shape change. Moreover, the wave arrives at the receiver with a delay. On the path propagating beyond the damaged area (path *T*_1_–*R*_3_), the first wave packet registered by the receiver is the same for the damaged plate and the plate without damage.

**Figure 11:**
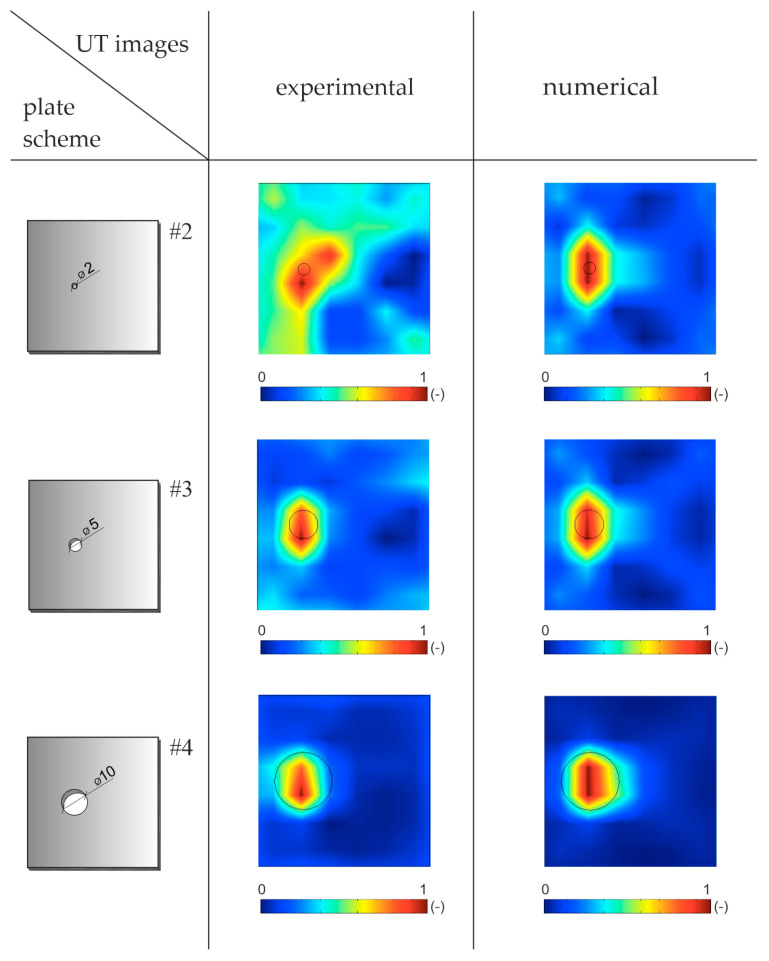
Ultrasonic tomography (UT) maps in plates with surface damage with the reference to the undamaged plate using TOF differences.

**Figure 12:**
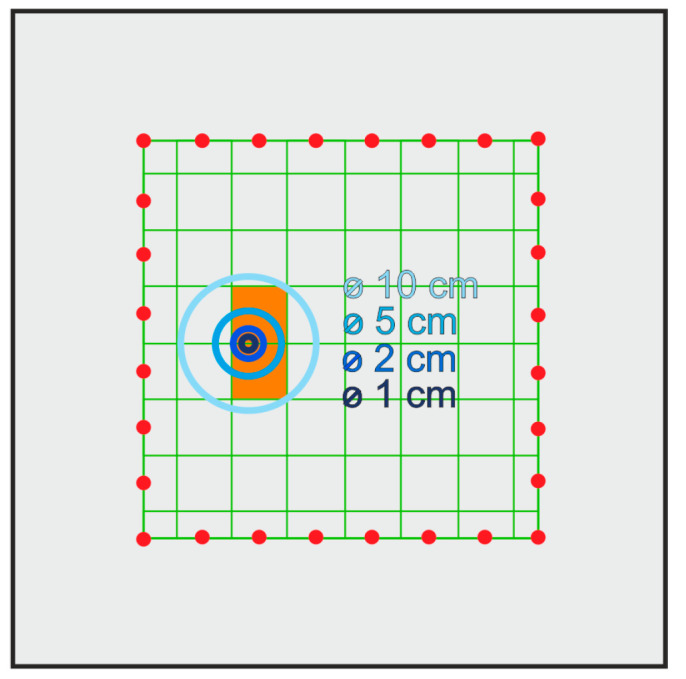
Location of damage with respect to the division of the element into pixels.

Figure 11 shows the tomographic velocity maps obtained based on differences in wave propagation TOF between the reference plate and the plate with growing damage. Each map was made on a scale from 0 to 1, where 1 indicates the highest difference between reference and current state, and 0 indicates their full compatibility. The location of the circular hole (Figure 11) was detected on the numerical images as regions with a reduced speed of wave propagation. These maps clearly indicate the location of the holes; however, it is not possible to precisely determine their size. This is due to the number of pixels into which the element was divided, as they define the image resolution. Regardless of the hole diameter, each defect lies on a combination of at least two pixels (see Figure 12). This area is indicated on tomography maps as a place with a reduced propagation velocity of ultrasonic waves. In the case of the UT images reconstructed for experimental signals, it can be noticed that they made it possible to determine the location of defects, regardless of their size. At the same time, it was not possible to assess the damage size, as in the case of the numerical results.

### 4.3. Influence of the Ray Tracing Technique

At the boundaries between regions with different velocities of wave propagation, an elastic wave can be refracted or reflected. In such a case, the wave rays may bend around a defect or other inclusion with a low wave propagation velocity [40]. The assumption that ultrasound waves propagate along straight paths from the transmitters to the receivers gives good results (cf. [41,42,43,44,45,46,47,48,49,50]). However, the quality of the tomography can be improved by using the actual ray path. In this study, ray tracing was performed utilizing the so-called hybrid approach, combining the ray bending methods with the network theory (e.g., [51]). The curved path is determined based on the values in individual pixels. The starting point is the image obtained for straight wave paths. Network theory creates a mesh of nodes on which the wave ray can travel. This mesh is usually denser than the pixel division. There are several ways to move from transmitter to specific receiver. The main purpose of the method is to find nodes through which the path must pass in order to reach the receiver as quickly as possible. To determine which path it is, Dijkstra’s algorithm was used. The solution is based on the velocity of wave propagation between two adjacent nodes and the distance between them. All nodes are divided into two groups. In the first group (group I) there are nodes for which the transit time is known. The second group (group II) contains the remaining elements. The schema of Dijkstra’s method includes the following steps (e.g., [11,52,53]):

1. Assign all nodes to group II and give them an infinite cost, except for the start node, whose cost is zero;
2. Choose the node from group II with the lowest value. Name it as *S* (start node) and transfer this node to group I;
3. Name as *N* (neighbour node) each node from group II that is connected to node *S*;
4. Calculate time travel between *S* and each *N* node using the equation:

(3) $$t(S)=\min(t(S);t(N)+t_{SN}),$$

(4) $$t_{SN}=\frac{d_{SN}}{\frac{\left(v_{S}+v_{N}\right)}{2}}$$

where *t*(*S*) denotes the travel time to reach node *S*, *t*(*N*) denotes the travel time to reach node *N*, *t_NS_* is the travel time between nodes *S* and *N*, *d_NS_* is the distance between nodes *S* and *N* and *v_S_* and *v_N_* are the values of the ultrasonic wave velocity in nodes *S* and *N*, respectively;

5. Repeat steps 2–4 until group II is empty.

Dijkstra’s algorithm assumes the checking of each node. When the fastest path was established, its straight sections were divided into an increasing number of straight but not collinear segments. The paths made in this way were naturally curved. The travel time was computed for each iteration, and the process ended when converging.

**Figure 13:**
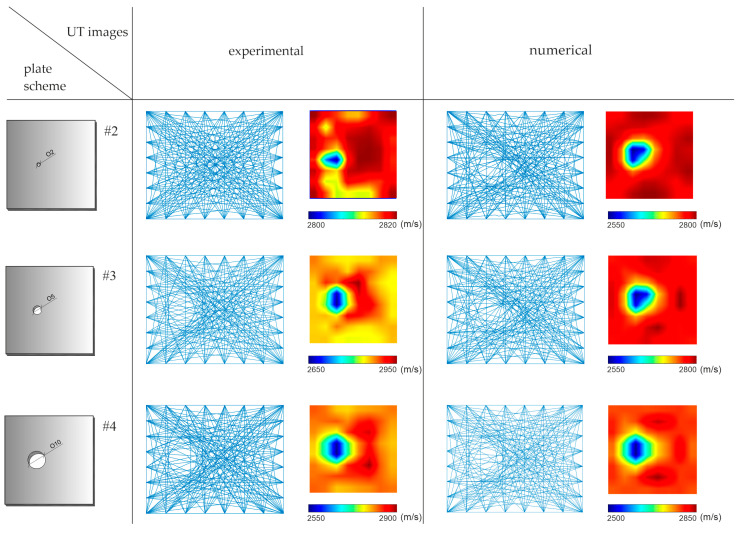
Ultrasonic tomography (UT) maps using direct travel time measurements with curved paths.

**Figure 14:**
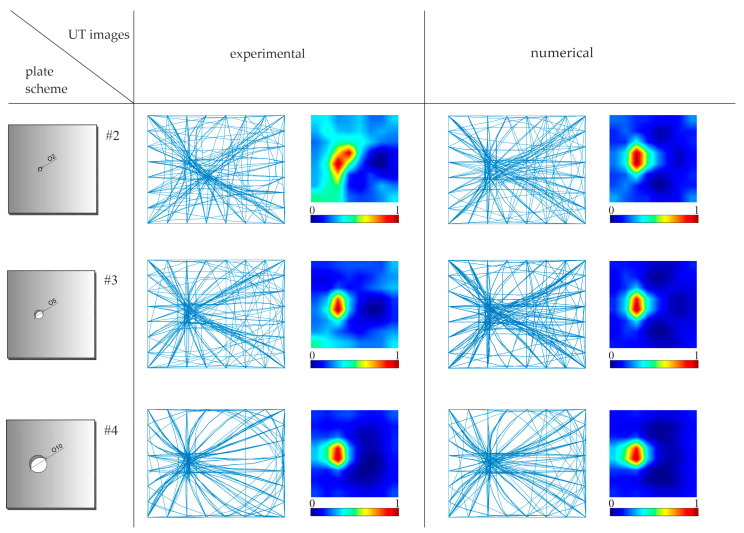
Ultrasonic tomography (UT) maps using the method of signal differences with curved paths.

Figure 13 and Figure 14 present experimental and numerical tomography maps created using the curved rays determined by the hybrid method. The analysis was carried out for the time of flight measured directly from transmitters to receivers (Figure 13) and by comparing the results between the current and reference state (Figure 14). The first step of the hybrid method is the preparation of a tomographic map for straight rays from transmitters to receivers. These maps are prepared both for the time of flight measured directly and for the differences in signals between the current and reference model. It is the starting point for which further ray bending iterations are carried out [52].

The tomographic velocity maps made on the basis of direct measurements (Figure 13) clearly indicate the location of the defects. They are visible as areas with smaller values of wave propagation velocity. Each of the tomographic images has its own individual scale, with values ranging from minimum to maximum velocity. Maps made for straight paths give satisfactory results. Simultaneously, the use of a hybrid method, combining the network method and the ray bending method, improved the results. In this case, the defect area was more concentrated. However, it was difficult to assess the extent of the damage based on these maps. Analysis of traced rays showed that the paths determined by the hybrid method bypassed the area of the defects.

Figure 14 shows tomographic velocity maps based on the comparison of the TOF between the undamaged plate and plates with growing defects. Values in the map cover the range from 0 to 1, where 1 indicates the highest measured difference between the map for undamaged and defective pieces, and 0 indicates their full compatibility. The circular hole in each plate is shown as an increased value, which means a significant difference in wave propagation velocity in this area. Curved rays are concentrated within the defect. In this case, the maps are similar to those prepared with the use of straight rays. However, the damaged area is only a little more concentrated. In this case, it was also impossible to estimate the damage size as in the case of straight rays.

### 4.4. Influence of the Pixel Grid Size

The existence and position of the surface growing damage in steel plates were determined based on the time of flight and UT reconstruction for both experimental and numerical data (see Figure 9, Figure 11, Figure 13 and Figure 14). However, the damage size assessment using the indicated methods did not reflect the actual dimensions of the hole. This was due to the size of the pixels into which the tested structure was divided. Such a division resulted from the number of transmitters and receivers used in the study. On two perpendicular edges, eight transmitters and eight receivers on opposite edges were used, which determined the division of the element into 64 pixels. The number of pixels affected the resolution of the obtained tomography image (cf. Figure 12).

**Figure 15:**
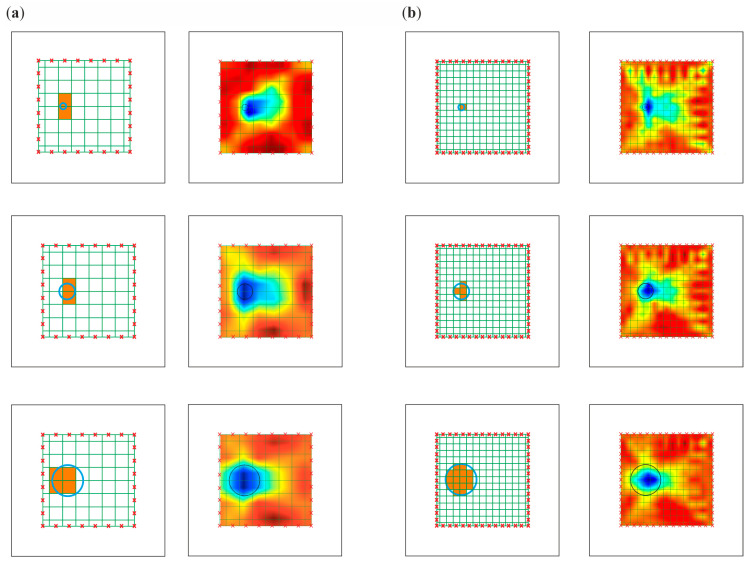
Pixel grid for the direct travel time measurement method: (**a**) division into 64 pixels; (**b**) division into 225 pixels.

**Figure 16:**
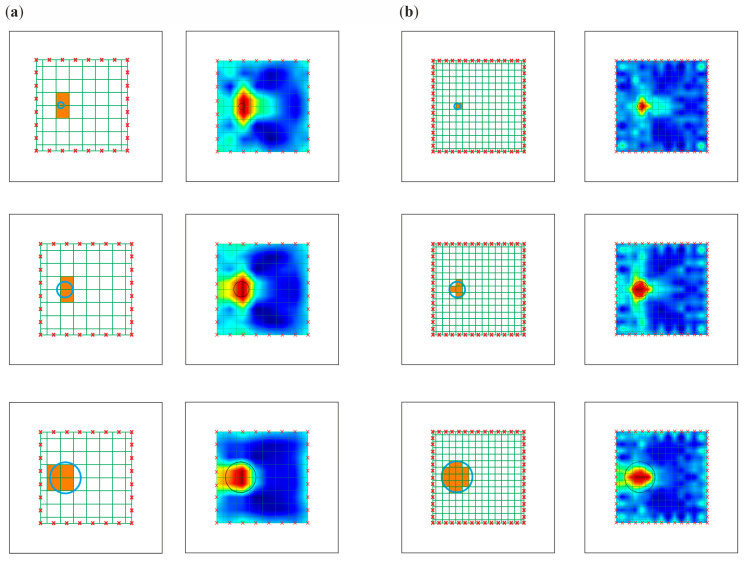
Pixel grid for travel time difference method (**a**) division into 64 pixels; (**b**) division into 225 pixels.

In order to improve the identification of the size of damage, the pixel mesh was refined. The split of each edge was increased from 8 to 15 elements, which gave a total number of 225 pixels. The dimension of a single pixel was 2.15 cm × 2.15 cm. The impact of the density grid was assessed for data obtained from numerical simulations. Figure 15 and Figure 16 show the tomography maps prepared for the elements divided into 64 and 225 pixels, respectively, using direct measurement of time of flight and difference in the TOF between the current state and the reference state. Orange colour marks the pixels for which the damage covers more than half of the area. In the case of a 64-cell mesh, the damage was concentrated within two pixels. The exception was damage with a diameter of 10 cm, which occupied 4 pixels. More significant differences in the number of pixels occupied by the damage are visible when the mesh was densified to 225 pixels.

The maps in Figure 15a and Figure 16a concern models divided into 64 pixels. The tomograms are very similar to each other, regardless of the damage size. The images were enhanced significantly by densifying the pixel grid (Figure 15b and Figure 16b). It was possible to estimate the damage size for such prepared tomograms. The size of the area with a lower velocities of wave propagation on these maps increased with the size of the defect.

### 4.5. Quantitative Analysis Using Error Coefficient

**Figure 17:**
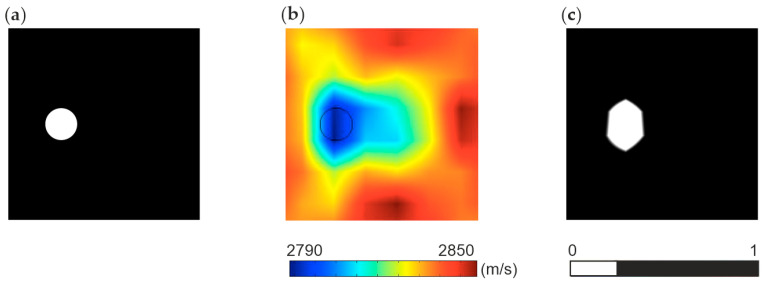
Error coefficient values for maps with reduced spectrum based on direct travel time measurement method: (**a**) reference model with a reduced spectrum, (**b**) topographic map with a full spectrum, (**c**) tomographic map with a reduced spectrum.

The accuracy of the tomographic reconstruction was quantified by using an error coefficient comparing the reference image with the obtained tomographic maps. Each of the prepared tomographic maps was replaced by a simplified model with a reduced spectrum, indicating high and low velocities as well as large and small differences. In the case of direct measurement, pixels with velocities below the 25th percentile of the maximum scale value are considered to be low-velocity indications and are assigned a value of 0, while others are assigned a value of 1. An example of spectrum reduction for direct measurement is shown in Figure 17. In the case of maps based on signal differences for an undamaged structure and a damaged structure, pixels with a difference above the 75th percentile of differences in a scale are assigned a value of 0, and the remaining pixels are assigned a value of 1.

**Figure 18:**
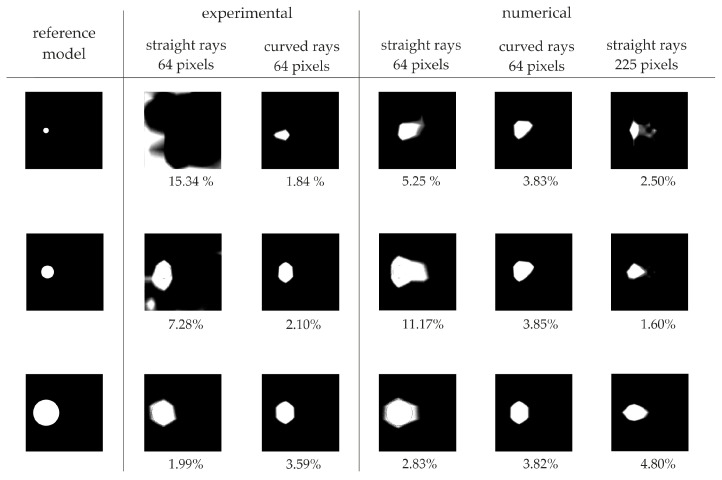
Error coefficient values for maps with reduced spectrum based on direct travel time measurement method.

**Figure 19:**
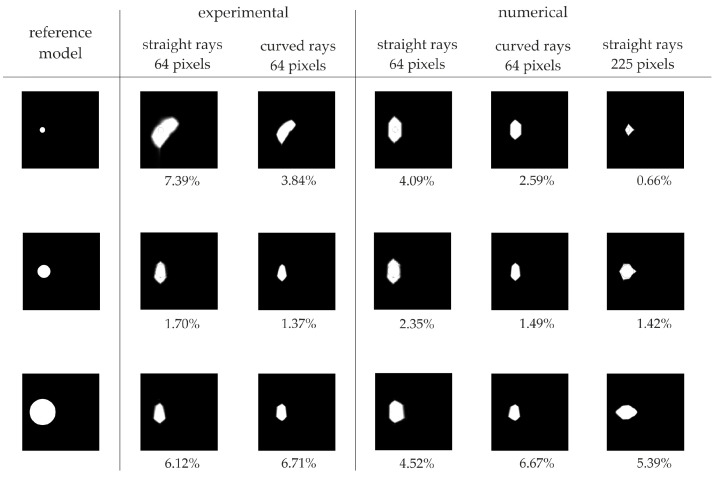
Error coefficient values for maps with reduced spectrum based on the travel time difference method.

Figure 18 and Figure 19 present maps with the reduced spectrum for direct measurement and for the travel time difference method, respectively. The first column indicates the reference model for which the defects have a value of 0, while the remaining area has a value of 1. The next two columns show the experimental results for straight and curved rays in the element divided into 64 pixels. Maps from numerical tests are summarized in columns 4–6 for straight and curved rays and for the element divided into 225 pixels. Below each map, the value of the error coefficient calculated by the following equation is shown:

(5) $$\gamma =\mathrm{mean}\left(\left|I^{j}-I^{j}{}_{ref}\right|\times 100\%\right),$$

where: $I^{j}$ is the value in i-th pixel of the considered ultrasound tomography map, and $I^{j}{}_{ref}$ denotes the value in the i-th pixel of the reference ultrasound tomography map.

In the case of defects with diameters of 2 and 5 cm, the error coefficient clearly indicates the improvement of the image quality, in the case of using the hybrid ray-tracing method and the densification of the pixel grid. The use of the hybrid method for experimental data improved the possibility of estimating the size of the defect with a diameter of 2 cm from 15.34 to 1.84% and 7.39 to 3.84%, respectively, for the direct measurement and for the comparative measurement of the damaged and undamaged model signals. For damage with a diameter of 5 cm, the improvement was from 7.28 to 2.10% and 1.70 to 1.37%. In the case of numerical tests, the error coefficient was calculated for the defect with a diameter of 2 cm as 5.25 and 3.83%, respectively using straight and curved radii in the direct measurement. At the same time, the value of the coefficient decreased to 2.50% in the case of the pixel grid density. The error coefficient value for the same measurements, but from the comparative method, was 4.09, 2.59 and 0.66%. In the case of a defect with a diameter of 5 cm, the use of the hybrid method improved the coefficient value from 11.17 to 3.85%. The error coefficient, when dividing the element into 225 pixels, was 1.60% for the direct measurement. The error coefficient for the comparative measurement and the 5 cm diameter defect is 2.35, 1.49 and 1.42% for straight wave paths, for curved paths and for a dense pixel grid, respectively. In the case of damage with a diameter of 10 cm, the value of the error coefficient slightly decreases. This is due to the accumulation of low-speed values in the case of applying both proposed methods to improve the image quality.

## 5. Conclusions

In tests carried out on steel plates, the use of ultrasound tomography to locate surface damage was assessed. Laboratory and numerical tests were carried out on four plates: one intact and three with surface damage of varying intensities. The performed ultrasound tomography was based on the reconstruction of the Lamb wave propagation velocity. Moreover, analyses were performed utilizing signal differences between the reference and defective plates. The conducted research allowed for the formulation of the following conclusions:

- Surface defects in the form of a circular hole were visualized effectively on tomograms as areas with reduced wave propagation velocity using both the TOF for the current state and the difference of the TOF between the current and reference state;
- The method comparing the TOF of ultrasonic waves propagating through a damaged and undamaged plate proved to be more effective, especially in the case of small defects;
- The apparent velocity of the waves propagating through the tested element decreased with the increase of the damaged area. At the same time, the value of standard deviation and coefficient of variation of wave propagation velocities increased;
- The use of curved wave paths improved the quality of the created ultrasonic tomography maps. However, at the same time, this approach did not allow assessing the damage size, which depends on image resolution, i.e., the number of pixels into which the examined area is divided;
- The course of curved paths was varied. In the case of discontinuities in the material, rays bypassed the place of the defect. However, when comparing the results of the damaged element with undamaged material, defects were detected as places of ray concentration;
- The possibility of assessing the damage size was related to the number of pixels into which the tested model is divided. The densification of the pixel grid made it possible to estimate the damage size more efficiently;
- The quantitative evaluation of the applied methods of densification of the pixel grid and the hybrid ray-tracing method was performed using an error coefficient. The coefficient clearly indicated the improvement in determining the size of the damage in the case of small defects with a diameter of 2 and 5 cm.

Lamb waves and their processing by the technique of ultrasound tomography proved to be an effective technique for imaging defects in thin plates. The presented approach is suitable for diagnosing defects in elements of real metal structures. Assessing the occurrence of damage can be particularly useful for monitoring plate structures for which the reference state is known, and the SHM system is designed to detect emerging and developing surface discontinuities.

## Figures and Tables

**Table 1:** Wave propagation velocities in steel plates for data from experimental tests.

Plate	*v*_min_ (m/s)	*v*_max_ (m/s)	Δ*v* = *v*_max_ − *v*_min_ (m/s)	*v*_avg_ (m/s)	SD (m/s)	CV (%)
#1	2789.50	2872.62	83.11	2835.33	22.75	0.80
#2	2764.14	2872.62	108.47	2807.85	25.27	0.90
#3	2746.01	2872.62	126.61	2821.63	27.47	0.97
#4	2555.47	2860.07	304.60	2791.01	53.29	1.91

**Table 2:** Wave propagation velocities in steel plates for data from numerical tests.

Plate	*v*_min_ (m/s)	*v*_max_ (m/s)	Δ*v* = *v*_max_ − *v*_min_ (m/s)	*v*_avg_ (m/s)	SD (m/s)	CV (%)
#1	2703.95	2807.84	103.89	2756.65	29.22	1.06
#2	2703.95	2807.84	103.89	2756.22	29.35	1.06
#3	2692.14	2844.99	152.85	2752.52	32.53	1.18
#4	2559.39	2891.45	332.06	2743.33	51.36	1.87

## Data Availability

The data presented in this study are available on request from the corresponding author.
